# Supplementary material for: Decision-Making in Implantology—A Cross-Sectional Vignette-Based Study to Determine Clinical Treatment Routines for the Edentulous Atrophic Mandible
Source: Int J Environ Res Public Health. 2021 Feb 8;18(4):1596. doi: 10.3390/ijerph18041596 (PMC7915536; doi:10.3390/ijerph18041596)
Supplement: Supplementary file 1 [file ijerph-18-01596-s001.zip › Zip/Figure S1.docx]

**Determinants of pre-implantological augmentation procedures – a case vignette study in the edentulous jaw**

| **1.**  **Here you will find case vignettes for the assessment of surgical treatment routines.** | | | |
| --- | --- | --- | --- |
| **Case 1** | | | |
| - This is a male patient, aged 55/ aged 75, wearing a mandibular denture. - **General anamnesis:** No systemic diseases. Currently not under medical treatment. - **Special anamnesis:** Patient is non-smoker / patient is a heavy smoker (20 cigarettes per day). - The patient wants a prosthetic restoration on implants in any case. - **Clinical findings:** Tooth 33 tooth loosening grade 3, PPD 6-7 mm, Tooth 43 tooth loosening grade 2, PPD 5-6 mm - **Course of the case:** Restoration in the upper jaw with 6 implants, sinus lift on both sides, incorporation of a removable prosthetic restoration approx. 1 year ago (locator abutments). - **Radiographic findings (orthopantomogram and CBCT):** mandibular atrophy - **Referrer’s request:** The referring dentist wishes to place a fixed prosthesis in the mandible on 4 implants. Teeth 33 and 43 are to be removed.   **Orthopantomogram**  **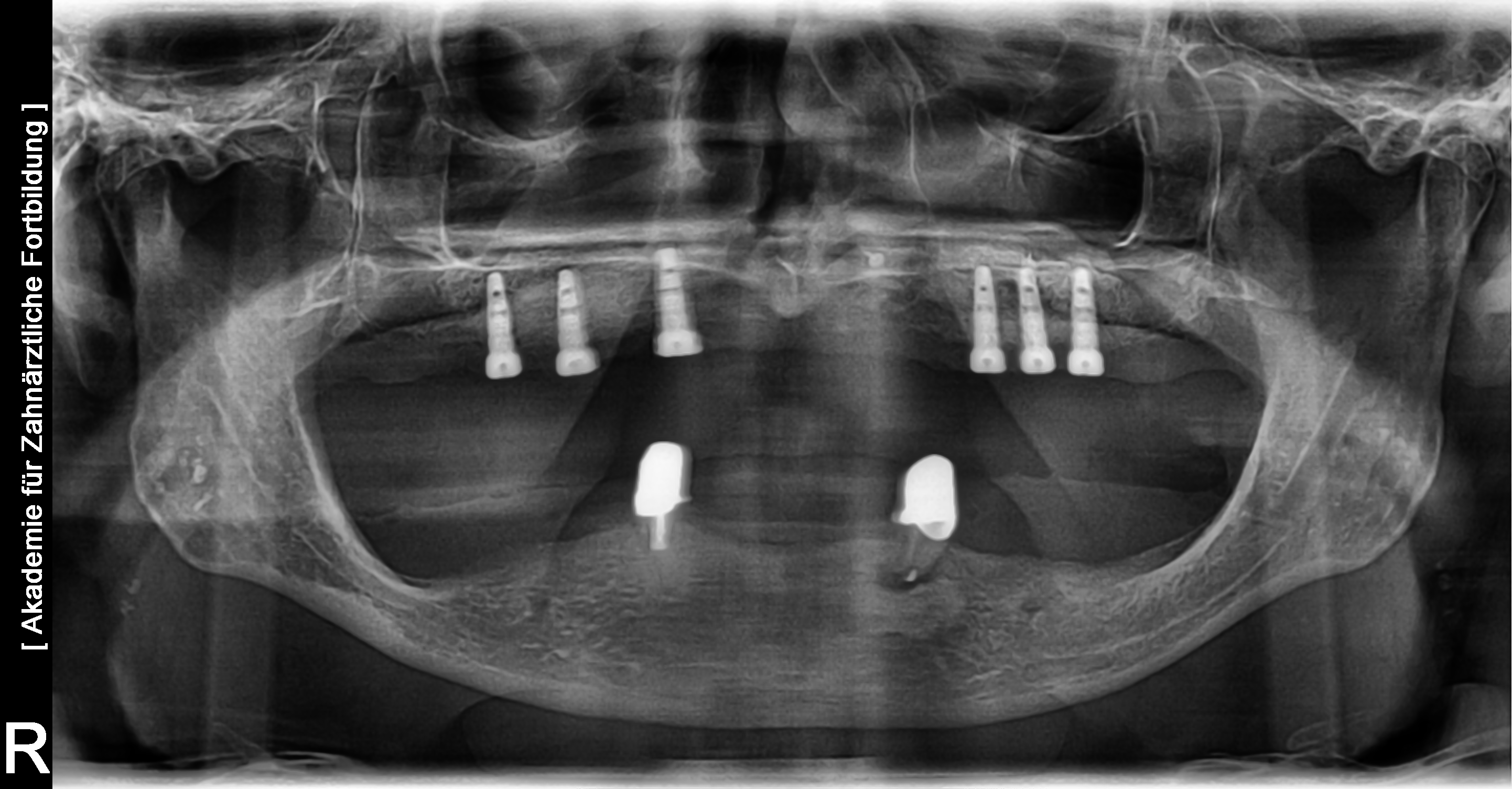**  **CBCT**  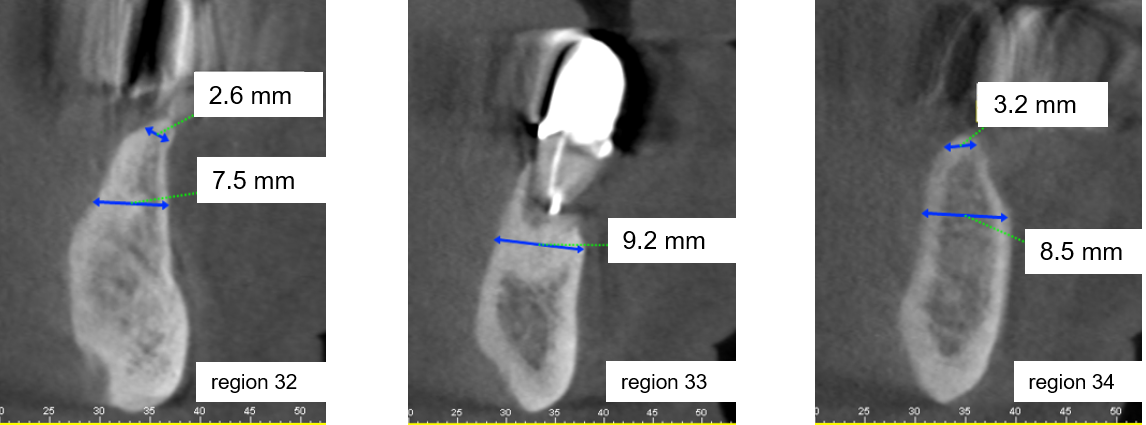  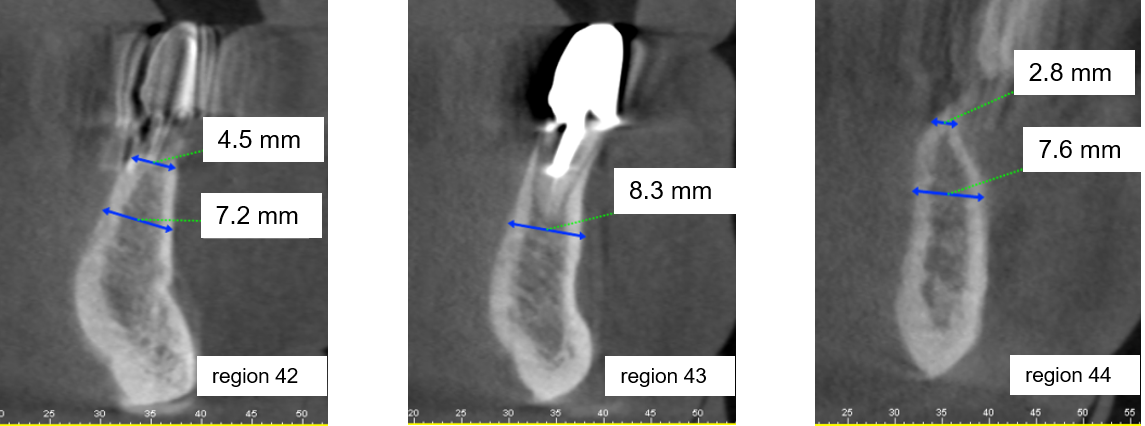 | | | |
| **Please analyze the records. How would you proceed surgically in this case?**  **Yes:** This option represents the therapy of my choice (please tick yes only once).  **Not at all:** This therapy is not possible  **Possibly:** I am considering this option. I will decide during surgery. You can select "Possibly" more than once. | **Yes** | **Not at all** | **Possibly** |
| Bone Split | 🞏 | 🞏 | 🞏 |
| Bone block | 🞏 | 🞏 | 🞏 |
| Augmentation with bone substitute material | 🞏 | 🞏 | 🞏 |
| Bone resection | 🞏 | 🞏 | 🞏 |
| No therapy | 🞏 | 🞏 | 🞏 |
| Other | ------------------------------------------------------------------ | | |

| **Case 2** | | | |
| --- | --- | --- | --- |
| - This is a female patient. Age 73 years. She is already edentulous for 10 years - **General anamnesis:** No systemic diseases. Currently not in the medical treatment / The patient has undergone radiotherapy in the neck area. The jaw bone was not in the radiation field. The salivary flow seems to be unaffected. - **Special anamnesis:** The patient cannot cope with her total denture in the mandible (repeated decubitus treatment). - The patient is very interested in a prosthetic restoration on implants. / The patient is rather anxious and repeatedly asks for an explanation of the procedure. - **Clinical findings:** little keratinized gingiva present - **Radiographic findings (orthopantomogram and CBCT):** lower and upper jaw atrophy - **Referrer’s request:** The referrer wishes to place a removable prosthesis in the mandible on 4 implants.     **Orthopantomogram**  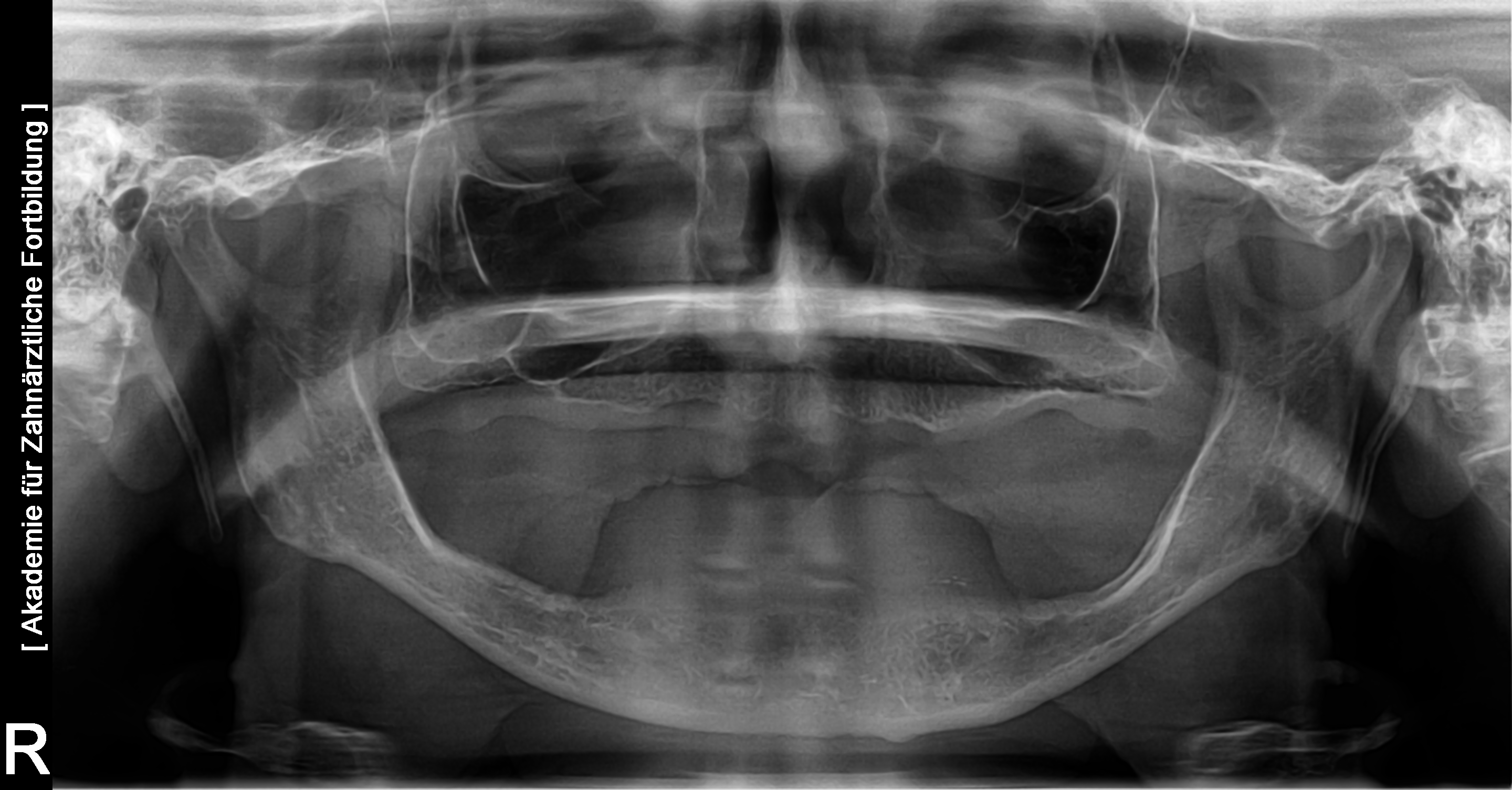    **CBCT**  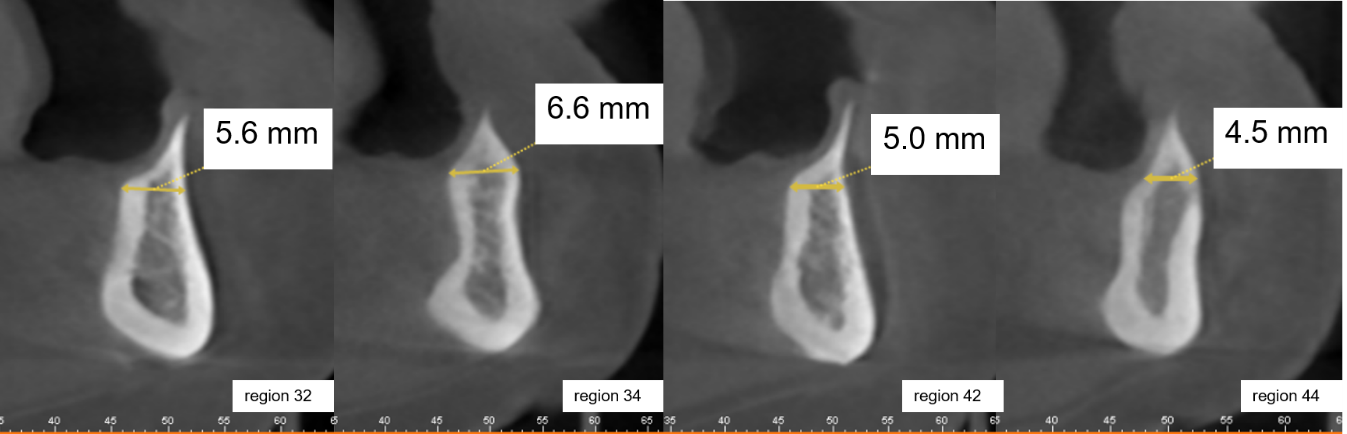 | | | |
| **Please analyze the records. How would you proceed surgically in this case?**  **Yes:** This option represents the therapy of my choice (please tick yes only once).  **Not at all:** This therapy is not possible  **Possibly:** I am considering this option. I will decide during surgery. You can select "Possibly" more than once. | **Yes** | **Not at all** | **Possibly** |
| Bone Split | 🞏 | 🞏 | 🞏 |
| Bone block | 🞏 | 🞏 | 🞏 |
| Augmentation with bone substitute material | 🞏 | 🞏 | 🞏 |
| Bone resection | 🞏 | 🞏 | 🞏 |
| No therapy | 🞏 | 🞏 | 🞏 |
| Other | ------------------------------------------------------------------ | | |

Commentary to the case vignettes:

The variable descriptors in the anamnesis were combined in this supplementary material. The variables are highlighted in red and green.
